# Draft Genome Sequence of *Exiguobacterium* sp. Strain BMC-KP, an Environmental Isolate from Bryn Mawr, Pennsylvania

**DOI:** 10.1128/genomeA.01164-15

**Published:** 2015-10-08

**Authors:** Peter Hyson, Joshua A. Shapiro, Michelle W. Wien

**Affiliations:** Department of Biology, Bryn Mawr College, Bryn Mawr, Pennsylvania, USA

## Abstract

*Exiguobacterium* sp. strain BMC-KP was isolated as part of a student environmental sampling project at Bryn Mawr College, PA. Sequencing of bacterial DNA assembled a 3.32-Mb draft genome. Analysis suggests the presence of genes for tolerance to cold and toxic metals, broad carbohydrate metabolism, and genes derived from phage.

## GENOME ANNOUNCEMENT

The genus *Exiguobacterium* is composed of organisms isolated from a wide range of environments often characterized by extreme conditions, such as heat or cold, high or low pH, or high salinity. The genus was first described by Collins et al. (1) in order to provide a new classification for a Gram-positive, non-spore forming, facultative anaerobic, alkaliphilic bacteria within the *Firmicutes*. *Exiguobacterium* species have been isolated from diverse environments, including permafrost (2, 3), soil (4), freshwater microbialites (5), a hyperalkaline spring (6), hot springs (7), plant rhizospheres (8), and glaciers in the Himalayas (9). *Exiguobacterium* spp. have also been implicated as opportunistic pathogens (10).

Here, we present the draft sequence of *Exiguobacterium* sp. strain BMC-KP cultured on a nutrient agar plate from swabs of the kitchen, bed, and a backpack in an apartment in Bryn Mawr, PA, as part of an undergraduate environmental sampling lab. The bacterium is Gram positive and appears yellowish orange on nutrient agar. It grows from 2.5 to 43°C and at pH levels from 6 to 10. The bacterium displays gelatinase, caseinase, and starch hydrolysis activities. Based on 16S rRNA gene phylogeny of *Bacillales* family XII Incertae sedis, *Firmicutes*, *Exiguobacterium* sp. BMC-KP falls within group I, which are organisms isolated from cold and temperate habits (11).

DNA was isolated using the Qiagen DNeasy kit. Whole-genome sequencing was performed on an Illumina MiSeq sequencer at the Indiana University Center for Genomics and Bioinformatics, using 300-bp paired-end reads constructed using the Illumina Nextera DNA library preparation kit, yielding 794,481 paired-end reads. Adapter contamination and low-quality sequences were removed using Trimmomatic 0.32.2 (12), and assembly was performed using the SPAdes Assembler 3.5.0 (13). The resulting assembly contigs were filtered for sequences with coverage of at least 40× and length >350 bp, resulting in a 3.32-Mb draft assembly consisting of 17 contigs with an *N*_50_ of 236,941 bp and median coverage of 120×.

Analysis using RAST (14) suggests the presence of genes encoding enzymes involved in the metabolism of many carbohydrates, including mannose, glycogen, beta-glucoside, and l-arabinose. The metabolism of l-arabinose was confirmed experimentally. The presence of proteins involved in stress tolerance, including cold shock, cadmium, and multidrug resistance, are also predicted. In addition, the sequence of BMC-KP contains phage DNA.

The genomic sequence of *Exiguobacterium* sp. BMC-KP allows further investigation of the genetic basis of the adaptation of the *Exiguobacterium* genus to a wide range of environmental conditions.

### Nucleotide sequence accession numbers.

This whole-genome shotgun project has been deposited in DDBJ/ENA/GenBank under the accession no. LGIW00000000; raw reads are available in the NCBI SRA archive under accession no. SRX1093385.
